# In vivo efficacy of artesunate–amodiaquine and artemether–lumefantrine for the treatment of uncomplicated falciparum malaria: an open-randomized, non-inferiority clinical trial in South Kivu, Democratic Republic of Congo

**DOI:** 10.1186/s12936-016-1444-x

**Published:** 2016-09-06

**Authors:** Marit de Wit, Anna L. Funk, Krystel Moussally, David Aksanti Nkuba, Ruby Siddiqui, Karla Bil, Erwan Piriou, Aldert Bart, Patrick Bahizi Bizoza, Teun Bousema

**Affiliations:** 1 Médecins sans Frontières (MSF), Plantage Middenlaan 14, 1018 DD Amsterdam, The Netherlands; 2 Manson Unit, Médecins Sans Frontières (MSF), 10 Furnival Street, London, EC4A 1AB UK; 3 Academisch Medisch Centrum, Amsterdam, The Netherlands; 4 Programme National de lutte contre le Paludisme, Kinshasa, South Kivu Democratic Republic of the Congo; 5 London School of Hygiene and Tropical Medicine, London, UK; 6 Radboud university medical center, Nijmegen, The Netherlands

**Keywords:** Uncomplicated malaria, Artesunate–amodiaquine, Artemether–lumefantrine, Child, Treatment efficacy, DR Congo, *Plasmodium falciparum*, DRC

## Abstract

**Background:**

Between 2009 and 2012, malaria cases diagnosed in a Médecins sans Frontières programme have increased fivefold in Baraka, South Kivu, Democratic Republic of the Congo (DRC). The cause of this increase is not known. An in vivo drug efficacy trial was conducted to determine whether increased treatment failure rates may have contributed to the apparent increase in malaria diagnoses.

**Methods:**

In an open-randomized non-inferiority trial, the efficacy of artesunate–amodiaquine (ASAQ) was compared to artemether–lumefantrine (AL) for the treatment of uncomplicated falciparum malaria in 288 children aged 6–59 months. Included children had directly supervised treatment and were then followed for 42 days with weekly clinical and parasitological evaluations. The blood samples of children found to have recurring parasitaemia within 42 days were checked by PCR to confirm whether or not this was due to reinfection or recrudescence (i.e. treatment failure).

**Results:**

Out of 873 children screened, 585 (67 %) were excluded and 288 children were randomized to either ASAQ or AL. At day 42 of follow up, the treatment efficacy of ASAQ was 78 % before and 95 % after PCR correction for re-infections. In the AL-arm, treatment efficacy was 84 % before and 99.0 % after PCR correction. Treatment efficacy after PCR correction was within the margin of non-inferiority as set for this study. Fewer children in the AL arm reported adverse reactions.

**Conclusions:**

ASAQ is still effective as a treatment for uncomplicated malaria in Baraka, South Kivu, DRC. In this region, AL may have higher efficacy but additional trials are required to draw this conclusion with confidence. The high re-infection rate in South-Kivu indicates intense malaria transmission.

*Trial registration* NCT02741024

**Electronic supplementary material:**

The online version of this article (doi:10.1186/s12936-016-1444-x) contains supplementary material, which is available to authorized users.

## Background

The Democratic Republic of Congo (DRC) is one of the five countries with the highest malaria burden in the world [1]. The eastern part of DRC is suffering from armed conflict and internal political instability. The conflict has caused massive suffering for civilians, with estimates of millions of deaths, directly or indirectly, as a result of the fighting [2, 3]. In this humanitarian crisis there are acute health needs, with limited access to humanitarian assistance and violations of basic rights and freedoms. A survey conducted in 2014 in South Kivu indicated a crude mortality rate (CMR) of 2.80 per 10,000 per day [95 % CI (2.40–3.28)] and an under-five mortality rate (<5MR) of 5.58 per 10,000 per day [95 % CI (4.60–6.76)]. Both of these mortality rates are more than two times above the emergency threshold [4].

**Fig. 1:**
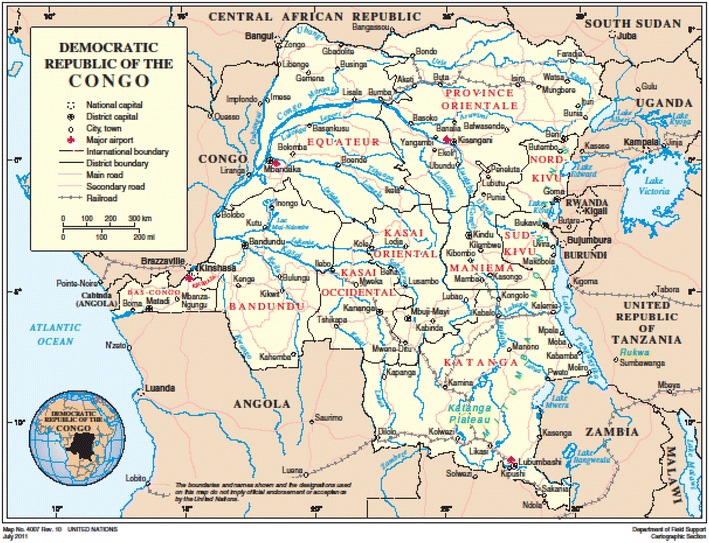
Map of the Democratic Republic of Congo

Médecins sans Frontières–Operational Centre Amsterdam (MSF-OCA or MSF) has been working in the provinces of North Kivu and South Kivu since the early 1990s and in Katanga since 2003 (Fig. 1). In South Kivu, MSF supports primary and secondary health care in the Baraka and Kimbi hospitals and in six health centres, and provides response to outbreaks and emergencies.

Malaria is holo-endemic in most of DRC, with seasonal fluctuations in transmission intensity in the east and south of the country. *Plasmodium falciparum* is the predominant species causing malaria in DRC [5]. Since 2003, MSF has introduced artemisinin-based combination therapy (ACT) in all programmes worldwide. Artesunate–amodiaquine (ASAQ) has been the first-line treatment for uncomplicated malaria in DRC since 2005. Artemether–lumefantrine (AL) is the second line treatment for uncomplicated malaria [6, 7]. Targeted distribution of long-lasting insecticide-treated bed nets (LLIN) to pregnant women and children hospitalized for severe malaria has been ongoing since 2003 in the MSF Baraka programme. The last mass LLIN distribution in this health zone was carried out in 2012.

**Fig. 2:**
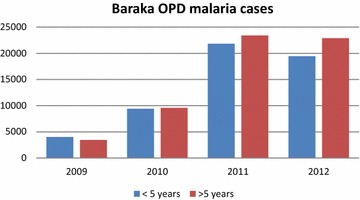
Confirmed malaria cases in Baraka project 2009–2012, DRC

Since 2009, MSF has seen an apparent rise in malaria incidence in most programmes in DRC. The National Malaria Control Programme (Programme National de Lutte contre le Paludisme, PNLP) has also observed an increase in malaria cases. In the Baraka programme, without any significant changes to the programme activities, the number of parasitologically confirmed uncomplicated malaria cases has risen from 7457 in 2009 to 44,317 cases in 2012 (Fig. 2). In order to investigate whether the observed increase in malaria over the years was associated with increased treatment failure rates, the efficacy of the first-line treatment, ASAQ was evaluated, and compared to that of AL in children aged 6–59 months with confirmed uncomplicated falciparum malaria.

## Methods

### Study design and site

An open-randomized non-inferiority trial comparing the efficacy of ASAQ to that of AL for the treatment of uncomplicated malaria, in children aged between six and 59 months, was conducted following the World Health Organization (WHO) protocols for surveillance of anti-malarial drug efficacy [8]. The study was carried out between October 2013 and December 2014 in the outpatient clinic of Baraka General Hospital and in the Health Centre of Baraka in South Kivu, DRC.

### Ethical considerations

The study was approved by the Ethical Review Board (ERB) of MSF on June 17th 2013 and by the Zone Chief Medical Officer (Médecin Chef de Zone) and the Provincial Medical Inspector (Médecin Inspecteur Provençal MIP) of South Kivu (N251/969/B.MIP/SK/2013). It was registered at ClinicalTrials.gov under number NCT02741024. Eligible patients were included in the study after an adult parent/caretaker gave written informed consent. If the parent/caretaker was illiterate, a literate witness was asked to sign next to the parent/caretaker’s fingerprint. Free health care for malaria and other illnesses is provided by MSF to the general population and was, therefore, also provided throughout the study follow-up period to all patients included in the study.

### Study population

Children aged between 6 and 59 months, presenting with fever (axillary temperature ≥37.5 °C) or reported history of fever in the last 24 h, and with a positive rapid diagnostic test (RDT) were enrolled in the study if they had: (1) a body weight ≥5 kg, (2) a slide confirmation of mono-infection with *P. falciparum* with an asexual parasite density between 2000 and 200,000/µl of blood, (3) an ability to swallow (crushed or dissolved) oral medication, (4) a high probability of respecting follow up visits, and (5) a signed informed consent by their adult (≥18 years old) parent/caretaker. Children were excluded if they presented with general danger signs according to the WHO protocol “Methods for surveillance of antimalarial drug efficacy” [8]. These included signs of severe/complicated malaria, including severe anaemia (Hb <5 g/dL), history of convulsions, and jaundice. Children were also excluded if they had severe acute malnutrition (indicated by a weight-for-height Z score (WHZ) of <−3 Z, a middle upper arm circumference (MUAC) of <115 cm, or bilateral oedema), a concomitant febrile or chronic illness, a known allergy to one of the study medications, or had received a full course of one of the artemisinin-based combinations under study in the previous 28 days.

### Sample size

Sample size calculations were based on the data that was available before October 2012. At that moment, studies determined the 42-day risk of recurrent parasitaemia due to recrudescence (treatment failure) in children to range from 0.9 to 6 % with AL and ASAQ [9–12]. Based on a conservative estimated risk of recurrent parasitaemia (due to recrudescence, PCR corrected) of 5 %, 120 patients per treatment arm would be needed to detect a difference in the risk of recrudescence between treatment arms of no greater than 7 % (one-sided type I error of 5, 80 % power) [13]. In order to account for undetermined PCR results, loss to follow up, withdrawal, and protocol violations, the total estimated sample size was increased by 20 % to 288 patients (144 per arm).

### Study procedure

All patients who were eligible for the study had a medical examination, and a MUAC and WHZ screening. A thick and a thin blood smear were performed and capillary blood was collected on a fast technology for analysis (FTA) card (Whatmann, UK). If the patient was eligible, informed consent was requested from the parent/caretaker after the explanation of the study by one of the study team members. Once the informed consent was obtained, patients were randomized to one of two treatment regimens according to which treatment had been randomly pre-allocated to that unique patient number. Treatment regimens consisted of: (1) ASAQ fixed dose (Winthrop Sanofi Aventis), given as 1 tab/day over three days (≥5– < 9 kg, 1 tab of 25 mg artesunate/67.5 mg amodiaquine base; ≥9–<18 kg, 50 mg artesunate/135 mg amodiaquine base), or (2) AL fixed combination (Coartem^®^, 20 mg artemether/120 mg lumefantrine, Novartis) given with milk twice daily over three days (≥5– <15 kg, 1 tab of 20 mg artemether/120 mg lumefantrine BD, ≥15–<25 kg, 2 tabs of 20 mg artemether/120 mg lumefantrine BD with fatty food) over 3 days. Drugs were given according to the manufacturer’s instructions and dosage. Study medication was imported and stored under strict regulations, ensuring control within the provided storage temperature and humidity ranges. The first dose of the study drug at day 0 (D0) was dispensed at the health clinic, and was taken by the child while under supervision of a study team member. For children who could not swallow tablets, AL tablets were dissolved, and ASAQ tablets were crushed in minimal amounts of water and dispensed with a spoon. After intake, the child was observed for 30 min. If the child vomited or spat out the medication within the monitoring period, a resting period of 15 min was observed before re-administering a repeat dose. If the repeat dose was also vomited within 30 min, the child was administered a rescue treatment (which was defined as the alternative treatment that the child was not randomized to) and was excluded from the study. Patients returned to the clinic on days 1 and 2 for observed administration of the study drug and a clinical assessment. In order to observe the intake of the evening doses of AL, patients randomized to receive AL were visited at home at around 8 h, 32 h and 56 h after the first dose. A follow up was scheduled for all patients on day 3, 7, 14, 21, 28, 35 and 42 for clinical assessment and blood smear evaluation.

If a clinical or parasitological treatment failure was observed, rescue treatment was initiated and a second blood sample was collected for PCR analysis. Caretakers were advised to present the child to the clinic at any time in case of illness. Patients who failed to return for scheduled follow up visits were traced immediately by home-visitors to minimize loss to follow up. Adverse events were evaluated by a physician and recorded at each visit.

### Treatment outcomes

Study endpoints were classified according to the WHO guidelines [8] as adequate clinical and parasitological response (ACPR), early treatment failure (ETF), late clinical failure (LCF) and late parasitological failure (LPF). Patients who were lost to follow-up, who withdrew their consent at any time before reaching a study endpoint, or who had a protocol violation (ex. wrongful inclusion by study staff, non-completion of full treatment course, administration of a study drug by a third-party, re-infection with a species other than *P. falciparum*) were not assigned an efficacy treatment outcome.

### Laboratory techniques

Initial testing for malaria infection was done on finger-prick blood using an RDT, the SD Bioline 05FK50 Malaria Ag P.f (Standard Diagnostics, Kyonggi, Republic of Korea). This RDT relies on detection of the Histidine Rich Protein 2 (HRP2), and is amongst the most sensitive according to the WHO product testing programme [14].

Thick and thin blood smears were stained with 10 % Giemsa for 15 min. Smears were read to 100 fields before they could be declared negative. Species were confirmed on the thin smear. Quantification of *P. falciparum* asexual parasitaemia on the thick smear was performed according to the WHO protocol “Methods for surveillance of antimalarial drug efficacy” [8]. Presence or absence of *P. falciparum* gametocytes was recorded.

Each slide was read, independently, by two qualified microscopists in the Baraka hospital laboratory, Parasite densities were calculated by taking a mean of the two counts. Blood smears with discordant results (differences in species diagnosis, in parasite density of >50 % or in the presence of parasites) were re-examined by a third microscopist who was blinded to the results of the first two, and parasite density was calculated by taking a mean of the two closest counts. In addition, external quality control of the slides was performed by blinded re-checking of 50 randomly selected malaria slides by an expert microscopist at the Epicentre Mbarare Research Base in Uganda.

Haemoglobin levels on D0 or during follow-up were determined using the HemoCue^®^ Hb 301 System (Ängelholm, Sweden), according to manufacturer’s instructions. PCR genotyping analysis was performed in order to distinguish true recrudescence (same parasite strain) from a newly acquired infection (different parasite strain) on capillary blood samples stored on FTA cards. The genotyping was performed at the Department of Medical Microbiology, at the Academic Medical Centre (Amsterdam, The Netherlands. PCR amplification of template DNA and analysis of *glurp*, *msp2* and msp1 alleles in pre- (enrolment) and post-treatment (failure) samples was performed according to the WHO protocol [15]. Pre- and post-treatment pairs with similar genotype were classified as recrudescence (true failure), and pairs with different genotypes were classified as re-infection according to WHO protocol “Methods and techniques for clinical trials on antimalarial drug efficacy: Genotyping to identify parasite populations” [15]. Species-specific PCRs were performed on samples with possible mixed re-infections according to Shokoples et al. [16].

### Data analysis

All data were double entered in the WHO global database for anti-malarial drug efficacy by two different epidemiologists, as well as in the worldwide anti-malarial resistance network (WWARN) database provided online [17]. Statistical analysis was performed using STATA version 13.1. Data was analysed per-protocol and by intention-to-treat. Kaplan–Meier survival curves were generated to determine the cumulative probability of recurrence-free survival over the 42 days follow-up. These probability estimates were compared using a log rank test. For the per-protocol analysis, patients who were lost to follow up or withdrawn were removed from the denominator. Patients were considered withdrawn from the PCR-corrected analysis if the PCR results were unclassifiable or if the results of PCR indicated that the failure was due to a mono re-infection with a *Plasmodium* species other than the *P. falciparum* (such as *Plasmodium vivax, Plasmodium malariae* or *Plasmodium ovale)*.

Chi square tests or Fisher’s exact tests were used to compare categorical data and Student’s *t* test was used for continuous data, as appropriate. The log rank test was used to compare Kaplan–Meier survival curves. p < 0.05 were considered statistically significant.

### Availability of data and materials

Data are available on request in accordance with MSF’s data sharing policy [18].

## Results

### Study trial profile and baseline characteristics

**Fig. 3:**
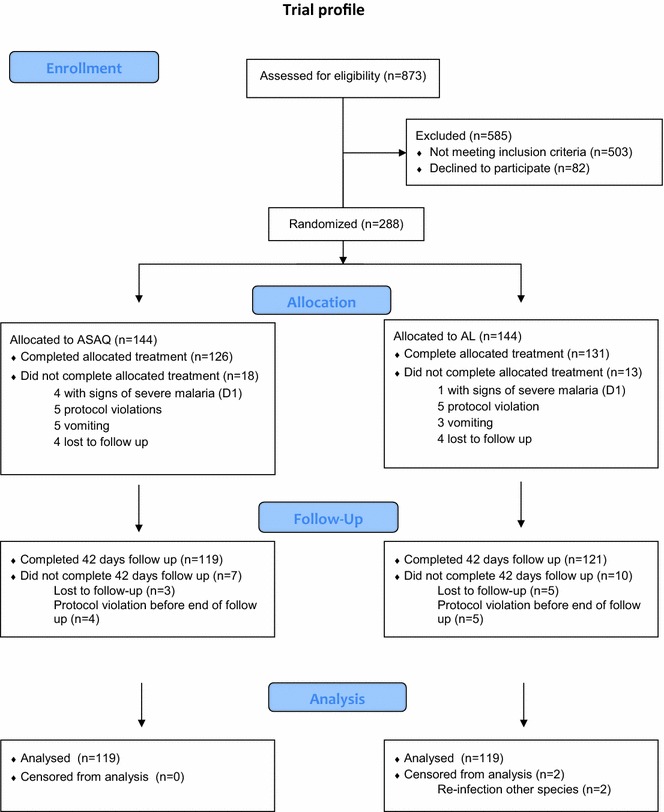
Trial profile efficacy study ASAQ–AL, Baraka, DRC

A description of the study outline is shown in Fig. 3. Out of 873 children screened for uncomplicated malaria during the study period, 585 (67.0 %) were excluded. The main reasons for exclusion were ACT intake in the last 28 days (118, 20.2 %), parasitaemia outside the study range (116, 19.8 %) or co-morbidities such as pneumonia or urinary tract infections (86, 14.7 %). 288 children were included in the study and were randomized to receive ASAQ or AL. 18 children (6.3 %) in the ASAQ group and 13 children (4.5 %) in the AL group did not complete the three days of allocated treatment, due to the following: developing (apparent) signs of severe malaria (n = 5 ETF, n = 3 wrongfully admitted to ward), a late realisation of wrongful enrolment (n = 10), having an observed treatment dose missed (n = 7), vomiting of at least one of the treatment doses twice (n = 5), a dosage error (n = 1). Of the children who received their full course of supervised treatment (257), 19 children (7.0 %) did not reach an analysable end result (censored), either due to being lost to follow up (n = 8), having had an anti-malarial administered by a 3^rd^ party (n = 9), or having been re-infected with a malaria species other than *P. falciparum* (n = 2).

The baseline characteristics of the patients enrolled in the study are presented in Table 1. Boys and girls were equally represented in the study population, with an average age of 31.5 months [(32.2 months for ASAQ (IQR 20.0–47.0) and 30.9 for AL (IQR 21.0–41.5)]. The average WHZ score was below international standards and similar in both treatment groups (–0.59 for ASAQ and −0.68 for AL) [19]. The average axillary temperature at D0 was higher in the AL group (39.02 °C, IQR 38.0–39.7) than in the ASAQ group (38.77 °C, IQR 38.4–39.8). No other statistically significant differences were observed between baseline characteristics for children randomized in both treatment arms.

**Table 1:** Baseline characteristics of included patients

	ASAQ (n = 144)	AL (n = 144)	p value
Age, in months	32.2 (20.0–47.0)	30.9 (21.0–41.5)	0.412
# Male (%)	65 (79)	65 (79)	1.000
Weight for height Z-score	−0.59 (−1.39 to 0.22)	−0.68 (−1.40–0.08)	0.484
Temperature in °C	38.8 (38.0–39.7)	39.02 (38.4–39.8)	0.049
Parasite density/µl at day 0	63,637 (15, 211–95, 962)	45,154 (14, 225–94, 746)	0.826
Log parasite density at day 0	10.5 (9.6–11.5)	10.4 (9.6–11.5)	0.935
Haemoglobin in g/dl at day 0	9.5 (8.5–10.8)	9.7 (8.7–10.8)	0.316

All data is presented as the mean with the interquartile range (IQR) in brackets, unless otherwise stated

### Treatment outcome results

Five children were classified as early treatment failures as per study protocol. All of these children had a parasitological improvement, but had either anaemia or jaundice that warranted admission to the hospital. They received intravenous artesunate according to the study protocol and the WHO guidelines for the treatment of malaria [20] and were withdrawn from the study.

Treatment efficacy estimates are presented in Table 2 for days 28 and day 42 after initiation of treatment. Before PCR genotyping, 23 (9.7 %) children (13/119 on ASAQ and 10/119 on AL) were classified as late clinical failure and 23 (9.7 %) children (14/119 on ASAQ and 9/119 on AL) as late parasitological failure on day 42. Furthermore, during the study period, two children who were on AL were re-infected with other species (*P. ovale*), as confirmed in weekly parasitological examinations; this was an involuntary protocol violation and these two children were censored in the final analysis. In total, 192 (80 %) children had an adequate clinical and parasitological response (92/119 for ASAQ and 100/119 for AL) at day 42. The proportion of recurring infection (including the two reinfections with other species) during the 42 days follow-up was higher in the ASAQ group (27/119, 22.7 %) compared to the AL group (21/119, 17.6 %), but this difference was not statistically significant. Similarly, cumulative failure on day 28 was not statistically significantly different between arms.

**Table 2:** Per-protocol crude/unadjusted and PCR-adjusted study endpoints at days 28 and 42 of patient follow-up. Patients with re-infections and individuals for whom the outcome could not be assessed by PCR were censored from the PCR adjusted analysis

Crude/no PCR	Day 28					Day 42				
	ASAQ (n = 119)		AL (n = 122)		p value	ASAQ (n = 119)		AL (n = 119)		p value
	n	%	n	%		n	%	n	%	
Late clinical failure	8	6.7	4	3.3	0.250	13	10.9	10	8.4	0.510
Late parasitological failure	10	8.4	6	4.9	0.277	14	11.8	9	7.6	0.273
Adequate clinical and parasitological response	101	84.9	112	91.8	0.093	92	77.3	100	84.0	0.189
Cumulative failure	18	15.1	10	8.2	0.093	27	22.7	19	16.0	0.189

PCR adjusted/corrected	Day 28					Day 42				
	ASAQ (n = 105)		AL (n = 111)		p value	ASAQ (n = 98)		AL (n = 101)		p value
	n	%	n	%		n	%	n	%	
Late clinical failure (recrudescence)	1	1.0	0	0	0.486	1	1.0	1	1.0	1.000
Late parasitological failure (recrudenscence)	3	2.9	0	0	0.113	5	5.1	0	0	0.027
Adequate clinical and parasitological response	101	96.2	111	100	0.038	92	93.9	100	99.0	0.049
Cumulative failure	4	3.8	0	0	0.054	6	6.1	1	1.0	0.062

The analysis of the treatment outcomes by intention-to-treat are shown in Additional file 1: Annexure 1

### PCR genotyping to determine re-infection and recrudescence

46 paired FTA filter paper samples were PCR genotyped, including all late clinical and parasitological failure samples. Following the PCR genotyping, 21 of the 27 recurrent parasitaemic episodes in the ASAQ arm (77.8 %) were classified as re-infections and 6 (22.2 %) as recrudescence. In the AL arm, 15 out of 19 samples (78.9 %) were classified as re-infections and 1 as (5.3 %) as recrudescence. There were no indications that treatment dosing was lower for the children experiencing treatment failure.

For three samples with recurring parasitaemia in the AL arm outcome could not be classified by PCR; these samples were censored at the last date of follow-up for which the child was still malaria negative. The proportions of children reaching each a study endpoint, before and after PCR adjustment, are presented in Table 2.

### Kaplan–Meier survival curves

**Fig. 4:**
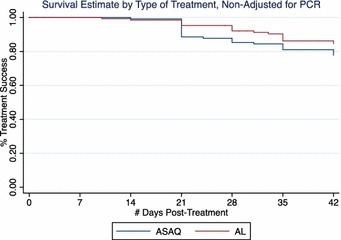
Kaplan–Meier survival estimates PCR-unadjusted. Treatment efficacy for ASAQ and AL was 85.3 and 92.1 % at day 28 (p = 0.0933), and 77.7 and 84.5 % at day 42 (p = 0.1605), respectively

**Fig. 5:**
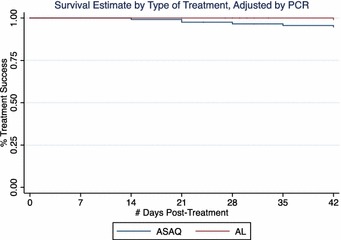
Kaplan–Meier survival estimates PCR-adjusted. Treatment efficacy for ASAQ and AL was 96.6 and 100 % at day 28 (p = 0.0406), and 94.7 and 99.0 % at day 42 (p = 0.0496), respectively

The cumulative probability of recurrence-free survival over the 42 days follow-up was determined by Kaplan–Meier survival analysis. The PCR un-adjusted curves shown in Fig. 4 indicate a non-significantly higher probability of remaining free of recurrent parasitaemia in the AL arm compared to the ASAQ arm (p = 0.16). After adjustment by PCR to distinguish between reinfection and recrudescence, the proportion of patients achieving ACPR at day 42 (treatment efficacy) was higher for the AL arm (99.0 %, 95CI 93.2–99.9) compared to the ASAQ arm (94.7 %, 95 CI 88.4–97.6), p = 0.0496 (Fig. 5). Regardless of the statistically significant differences between study arms, the difference in ACPR by day 42 after initiation of treatment was within the margin of non-inferiority.

### Parasite clearance

A relatively large proportion (13.7 % on ASAQ, 20.0 % on AL) of children still had parasites on day 2 of follow-up. This proportion fell to below 5 % in both arms on day 3. There was no significant difference in the proportion of parasites at D2 and D3 between the two treatment arms (Table 3).

**Table 3:** Parasite clearance on Days 2 and 3 of treatment

	ASAQ	AL	p value
Patients with parasite positivity at D2	18 (13.7 %)	27 (20.0 %)	0.173
Patients with parasite positivity at D3	2 (1.5 %)	6 (4.4 %)	0.167

### Treatment safety

There were no serious adverse events during the period of the study. However, minor adverse events occurred in both treatment arms during the treatment period (days 0–2). The adverse event most reported was asthenia (weakness, lack of energy and strength) for both arms. Children receiving ASAQ reported significantly more asthenia and anorexia (loss of appetite) than children receiving AL (Table 4).

**Table 4:** Adverse events = classified as related to the drug, for all patients during the study period

	ASAQ (n = 144)	AL (n = 144)	Total	p value
Asthenia	61 (42.4 %)	13 (9.0 %)	74 (25.7 %)	<0.001
Anorexia	27 (18.8 %)	12 (8.3 %)	39 (13.5 %)	0.010
Vomiting	11 (7.6 %)	10 (6.9 %)	21 (7.3 %)	0.821
Cough	5 (3.5 %)	7 (4.9 %)	12 (4.2 %)	0.555
Abdominal Pain	6 (4.2 %)	2 (1.4 %)	8 (2.8 %)	0.151
Diarrhoea	4 (2.8 %)	2 (1.4 %)	6 (2.1 %)	0.409
Itching	3 (2.1 %)	1 (0.7 %)	4 (1.4 %)	0.314
Nausea	1 (0.7 %)	1 (0.7 %)	2 (0.7 %)	1.000

## Discussion

The results of this study indicate that both study drugs retain adequate efficacy (PCR corrected cure rate at day 42 of 94.7 % for ASAQ and 99.0 % for AL) in the treatment of uncomplicated *P. falciparum* malaria in children aged six to 59 months in the study setting. In 2004, a similar study in DRC (Pool Region) showed comparable results (98.5 % efficacy of ASAQ and 100 % efficacy of AL at 28 days) [21]. This was also the result of the efficacy study by Singana et al. [22] in Owando in Congo-Brazzaville done in 2012–2103 (efficacy of ASAQ 98.0 % and of AL 100.0 % at 28 days, after PCR correction). The 4ABC study group, performed in a comparable population and using the same methodology, showed a similar efficacy rate between 2007 and 2009 over seven sub-Saharan African countries (96.8 % efficacy of ASAQ and 95.5 % efficacy of AL at 28 days) [23]. In the 4ABC study, ASAQ was also shown to be effective in Eastern Africa, whereas other studies had previously casted doubts over its efficacy in this region [24, 25].

The high efficacy of both ASAQ and AL in this region is reassuring. WHO only recommends a switch in the first line treatment if the efficacy falls below 90 % [26]. Although this study confirms that both artemisinin-based combinations retain good efficacy in this part of DRC, it does, however, demonstrate high re-infection levels. After PCR correction, this study showed that, over 6 weeks, 21/119 children (17.6 %) of those who were on ASAQ and 15/119 (12.6 %) of the children in the AL treatment group (p = 0.29) were re-infected within 42 days.

Whilst re-infections do not indicate failing anti-malarial drugs, they are of public health relevance and pose a considerable burden to the population and health systems. The results of this study seem to support recent findings that the period over which lumefantrine and amodiaquine provide post-treatment protection is 10–14 days [26–28]. However, considering the public health impact of frequent malaria infections in infants, it would be useful to do further analysis into the specific protective period for each of ASAQ and AL in this setting. It is also worth considering if the use of an ACT with a longer protective half-life, such as dihydroartemisinin–piperaquine, may be beneficial to prevent malaria episodes in the study population, exposed to intense malaria transmission [27–29].

In this study’s setting children will normally receive ASAQ each time they have a malaria infection. Reassuringly, Yeka et al. [29] showed that repeated treatment using both ASAQ and AL is effective, safe and well-tolerated in children under 5 years of age and in a similar context [29].

With regards to tolerability, there were no serious adverse events registered during the trial. Asthenia and anorexia were the most commonly recorded minor adverse events with a marked difference between the two drugs. It was shown by the results of this study that AL was better tolerated. During the study it was observed that the parents/caretakers would prefer that their child be randomized to AL rather than ASAQ as the former was perceived as more tolerable by the children. In fact, the chance to be randomized to receive AL seemed to be a major driving force to participate in the study. Although asthenia and anorexia may be classified as minor adverse effects, it can have a negative effect on adherence, and thus prevent a complete cure.

There was a relatively high proportion (67 %) of children excluded from the study at the time of screening. There were two major reasons for exclusion: the high number of children with co-morbidities, and the high number of children who had taken ACT in the previous 28 days (either from a clinic or from a private vendor). In the children with co-morbidities, it is not clear what relation malaria had to their clinical illness; the children could be presenting with clinical illness due to malaria, or have symptoms primarily from their co-morbidity but happen to have malaria parasites. This is a dilemma that is faced often in the field, and may lead to an overestimation of the burden of malaria. Furthermore, an RDT for malaria is often performed as a first means of triage for fever, and when the test is positive, there is the risk that the child may not be adequately examined for other illnesses. In this study, 49 of the 873 (5.6 %) children with a positive RDT (based on HRP2 detection) had a negative thick smear. This could be the result of circulating HRP2 from a previous, well treated malaria infection. This is expected considering that Grandesso et al. (internal communication, Clinical Trial.gov Identifier NCT01325974) have shown that over 50 % of HRP2 RDTs remain positive more than 6 weeks after adequate treatment of a malaria infection.

## Conclusion

ASAQ is still effective as a treatment for uncomplicated malaria in Baraka, South Kivu, DRC, 9 years after its introduction as a first-line treatment. There is no evidence that ASAQ is inferior to AL in this setting with the non-inferiority margin of 7 % that was set for the current study. AL may have higher efficacy but additional trials are required to draw this conclusion with confidence. There is a high rate of re-infection in South-Kivu within six weeks of adequate treatment of either ASAQ or AL, indicating intense malaria transmission. AL was better tolerated by patients than ASAQ. Although earlier estimates of anti-malarial drug efficacy are unavailable for the current population, the high efficacy of ASAQ makes it unlikely that declining drug efficacy is a reason for the apparent increase in malaria cases observed in the region.

## Additional file

10.1186/s12936-016-1444-x Intent-to-treat crude/unadjusted and PCR-adjusted study endpoints at days 28 and 42 of patient follow-up.
