# Supplementary material for: In vivo efficacy of artesunate–amodiaquine and artemether–lumefantrine for the treatment of uncomplicated falciparum malaria: an open-randomized, non-inferiority clinical trial in South Kivu, Democratic Republic of Congo
Source: Malar J. 2016 Sep 6;15(1):455. doi: 10.1186/s12936-016-1444-x (PMC5013565; doi:10.1186/s12936-016-1444-x)
Supplement: Supplementary file 1 — 10.1186/s12936-016-1444-x Intent-to-treat crude/unadjusted and PCR-adjusted study endpoints at days 28 and 42 of patient follow-up. [file 12936_2016_1444_MOESM1_ESM.docx]

**Annex 1:** Intent-to-treat crude/unadjusted and PCR-adjusted study endpoints at days 28 and 42 of patient follow-up.

|  | **Day 28** | | | | | **Day 42** | | | | |
| --- | --- | --- | --- | --- | --- | --- | --- | --- | --- | --- |
|  | **ASAQ (n=144)** | | **AL (n=144)** | | **p-value*** | **ASAQ (n=144)** | | **AL (n=144)** | | **p-value*** |
| **Crude/ No PCR** | **n** | ***%*** | **n** | ***%*** |  | **n** | ***%*** | **n** | ***%*** |  |
| Early Treatment Failures | 3 | *2.1* | 2 | *2.1* | 1.000 | 3 | *2.1* | 2 | *1.4* | 1.000 |
| Late Clinical Failure | 8 | *5.6* | 4 | *2.8* | 0.377 | 13 | *9.0* | 10 | *6.9* | 0.514 |
| Late Parasitological Failure | 10 | *6.9* | 6 | *4.2* | 0.303 | 14 | *9.7* | 9 | *6.3* | 0.277 |
| Adequate Clinical and Parasitological Response | 101 | *70.1* | 112 | *77.8* | 0.140 | 92 | *63.9* | 100 | *69.4* | 0.317 |
| Withdrawal | 19 | *13.2* | 15 | *10.4* | 0.465 | 19 | *13.2* | 18 | *12.5* | 0.860 |
| Lost to follow-up | 3 | *2.1* | 5 | *3.5* | 0.723 | 3 | *2.1* | 5 | *3.5* | 0.723 |
| Cumulative Failure | 21 | *14.6* | 12 | *8.3* | 0.096 | 30 | *20.8* | 21 | *14.6* | 0.165 |
|  | | | | | | | | | | |
|  | **Day 28** | | | | | **Day 42** | | | | |
|  | **ASAQ (n=144)** | | **AL (n=144)** | | **p-value*** | **ASAQ (n=144)** | | **AL (n=144)** | | **p-value*** |
| **PCR adjusted/ corrected** | **n** | ***%*** | **n** | ***%*** |  | **n** | ***%*** | **n** | ***%*** |  |
| Early Treatment Failures | 3 | *2.1* | 2 | *1.4* | 1.000 | 3 | *2.1* | 2 | *1.4* | 1.000 |
| Late Clinical Failure** | 1 | *0.7* | 0 | *0* | 1.000 | 1 | *0.7* | 1 | *0.7* | 1.000 |
| Late Parasitological Failure** | 3 | *2.1* | 0 | *0* | 0.247 | 5 | *3.5* | 0 | *0* | 0.060 |
| Adequate Clinical and Parasitological Response | 101 | *70.1* | 111 | *77.1* | 0.181 | 92 | *63.9* | 100 | *70.9* | 0.317 |
| Reinfection | 14 | *9.7* | 8 | *5.6* | 0.183 | 21 | *14.6* | 15 | *10.4* | 0.285 |
| Withdrawal | 19 | *13.2* | 18 | *12.5* | 0.860 | 19 | *13.2* | 21 | *14.6* | 0.733 |
| Lost to follow-up | 3 | *2.1* | 5 | *3.5* | 0.723 | 3 | *2.1* | 5 | *3.6* | 0.723 |
| Cumulative Failure | 7 | *4.9* | 2 | *1.4* | 0.173 | 9 | *6.3* | 3 | *2.1* | 0.138 |

*****p-value was determined by chi2 test (if n>5) or fishers exact test (if n<=5) **Recrudescence

Five children were classified as early treatment failures as per study protocol. All of these children had a parasitological improvement, but had either anaemia or jaundice that warranted admission to the hospital. It is unlikely that this is due to failing efficacy of the study drugs. They received intravenous artesunate according to the study protocol and the WHO guidelines for the treatment of malaria[19] and were withdrawn from the study.
